# Does Quality of Life in Survivors of Surgery for Acute Left-Sided Infective Endocarditis Differ from Non-Endocarditis Patients?

**DOI:** 10.3390/microorganisms11041058

**Published:** 2023-04-18

**Authors:** Alejandro Fernández-Cisneros, Aida Andreu, Marta Hernández-Meneses, Jaume Llopis, Elena Sandoval, Daniel Pereda, Jorge Alcocer, Manuel Castellá, Jose M. Miró, Eduard Quintana

**Affiliations:** 1 Cardiovascular Surgery Department, Hospital Clinic—IDIBAPS, University of Barcelona, 08036 Barcelona, Spain; alejandro.fernandez.cisneros@gmail.com (A.F.-C.);; 2 Infectious Diseases Department, Hospital Clinic—IDIBAPS, University of Barcelona, 08036 Barcelona, Spainjosemaria@miromoreno.org (J.M.M.); 3 Department of Genetics, Microbiology and Statistics, Faculty of Biology, University of Barcelona, 08036 Barcelona, Spain; 4 CIBERINFEC, Instituto de Salud Carlos III, 28029 Madrid, Spain

**Keywords:** infective endocarditis, quality of life, short form 36-item health survey, surgery for infective endocarditis

## Abstract

Surgery for left-sided infective endocarditis (IE) has been demonstrated to improve patients’ survival rates but information about quality of life (QoL) after surgery is scarce. The aim of this study was to assess the postoperative outcomes and QoL after surgery for IE patients compared to patients undergoing cardiac surgery for non-IE indications. Adult patients with definite acute left-sided IE were matched 1:1 to patients who underwent cardiac surgery for non-endocarditic purposes from 2014 to 2019. QoL was assessed using the SF-36 survey at the last follow-up. A total of 105 patients were matched. The IE group had higher rates of preoperative stroke (21% vs. 7.6%, *p* = 0.005) and higher stages of NYHA class (*p* < 0.001), EuroSCORE II (12.3 vs. 3.0, *p* < 0.001) and blood cell count abnormalities (*p* < 0.001). The IE group had higher incidence of low cardiac output syndrome (13.3% vs. 4.8%, *p* = 0.029), dialysis (10.5% vs 1.0%, *p* = 0.007) and prolonged mechanical ventilation (16.2% vs. 2.9%, *p* = 0.002) after surgery. At the last follow-up, subcomponents of the SF-36 QoL survey were not different between the groups. Patients who underwent cardiac surgery for IE demonstrated a higher risk profile with a higher rate of postoperative complications. Once recovered from the acute phase of the disease, the reported QoL at follow-up was comparable to that of matched cardiac patients operated for non-IE purposes.

## 1. Introduction

A substantial proportion of patients with infective endocarditis (IE) will need surgery in the acute phase of disease to facilitate infection control, restore valve function and/or prevent systemic embolism [1,2]. Surgery for IE has been associated with a decreased risk of mortality in all age groups [3]. The treatment for these IE patients is challenging and requires an individualized approach through a multidisciplinary endocarditis team [4,5]. Mortality during the acute phase of IE remains high, ranging from 13% to 25%, and after hospital discharge, up to 11.2% of the patients will die within the first year [6]. The outcomes of surgery have been traditionally assessed in terms of perioperative mortality and complications. However, due to the inherent systemic involvement of the disease, therapies addressed to grant survival may ultimately affect the long-term quality of life (QoL). This impact over QoL and burden of global functioning after IE has been seldomly explored in the existent literature, especially in the surgical population [7,8,9]. One of the available tools to assess QoL is the 36-Item Short Form Survey (SF-36) which evaluates the patients’ physical, psychological and social performance [10,11]. The SF-36 questionnaire is widely used worldwide and has proved to be suitable for the evaluation of QoL in multiple medical conditions and in cardiac surgery patients [12]. 

The focus of this study was to assess the QoL in patients who underwent surgery for acute left-sided IE and determine the potential impact of surgery over QoL compared to non-IE post-operated cardiac patients.

## 2. Materials and Methods

### 2.1. Design

Single-center case-control study of patients who underwent cardiac surgery with a diagnosis of acute left-sided IE from May 2014 to December 2021. Post-operated cardiac patients for IE purposes were matched head-to-head to control patients at a 1:1 ratio. Controls were defined as adult patients who underwent elective cardiac surgery for non-IE purposes in the same institution during the same period. This study was approved by the institutional board of the Hospital Clínicof Barcelona (HCB/2021/0238).

### 2.2. Patient Selection and Data Collection

All consecutive patients with a definite diagnosis of acute left-sided IE who underwent surgical treatment in the acute phase of disease were included in this study. The IE diagnosis was made in all cases following the modified Duke criteria and the consensus of the IE team [5,13]. The surgical indications followed the current IE ESC guidelines 2009 and 2015 and were agreed by the endocarditis team [5,14,15]. The control group of this present study comprised adult patients who underwent elective cardiac surgery for non-IE purposes. 

Control cases were selected from the departmental cardiovascular database. Matching criteria included sex, age (±6 years), valve involved and concomitant procedures such as coronary artery bypass graft surgery (CABG), aortic surgery or tricuspid surgery. Exclusion criteria were urgent or emergent surgery, congenital heart defects, aortic dissection, hypertrophic obstructive cardiomyopathy and previous IE. Matching was performed 1 to 1 according to first-matching patient who fulfilled similar characteristics for a given case. 

Initially, the IE group consisted of 176 patients. During follow-up, 37 patients died, and 18 patients could not be contacted or were excluded because of inability to respond the SF-36 questionnaire. This latter group included patients with inability to be contacted (6), transferred to their referring institution (3), refusing to participate (3), with severe neurological impairment (2), in a social assistance program (2), admitted to the hospital while the study was conducted (1) and with a language barrier (1). Thus, a total of 121 patients were eligible to participate in the study. For these patients, matching with a control case was performed following the aforementioned criteria. Due to the high technical complexity, 9 patients could not be matched to a control with similar characteristics and, in consequence, were excluded from the study. In the control group, 10 of the first matched patients died before being offered the questionnaire, and 8 could not be contacted or were excluded due to clinical reasons, including inability to contact them (3), dementia (2), additional catheter-based intervention (2) and being elderly at hospice care (1). Therefore, new controls were included, and 11 patients could be matched. Finally, 105 cases were matched and represented our study population.

### 2.3. Clinical Data

Pre-operative characteristics and post-operative data were harvested from medical records and from the departmental database. Pre-operative characteristics included demographic data, baseline characteristics and related health conditions, laboratory parameters and echocardiographic data. The functional class was evaluated with the New York Heart Association (NYHA) dyspnea classification, and preoperative risk assessment was performed with EuroSCORE II [16]. The intraoperative variables included cardiopulmonary bypass parameters, associated procedures and type of valvular substitute. The evaluation of outcomes included the collection of post-operative complications and the assessment of quality of life at the last follow-up.

### 2.4. SF-36 Questionnaires

Questionnaires were administered at the last follow-up in person in the outpatient clinic, by phone or via email in both groups. Quality of life was assessed using the Spanish version of the 36-Item Short Form Survey (SF-36) [10]. The SF-36 consists of 36 items which evaluate 8 subcomponents comprising physical functioning (PF), role physical (RP), bodily pain (BP), general health (GH), vitality (VT), social functioning (SF), role emotional (RE) and mental health (MH). The raw scores from the eight subcomponents were standardized to a 0–100 score, where 0 was the lowest possible scoring, and 100 was the maximum possible scoring. Average scores and 95% confidence interval (95% CI) were calculated for each subcomponent. In addition, 2 component summary dimensions were calculated using the relative weights for each subcomponent according to Vilagut and cols [17]. Those dimensions were summarized as physical component summary (PCS) and mental component summary (MCS) [18]. The questionnaires were administered only by two investigators (AFC and AA), and the graduation of the possible answers was agreed prior to recollecting the data to minimize investigators’ bias.

### 2.5. Statistical Analysis

Continuous variables are described as median and interquartile range (IQR), and categorical variables are described as frequencies and proportions. The IE and non-IE groups were baseline-compared to determine if both groups were similar with the U-Mann–Whitney test for continuous data or the chi-squared test for categorical data, as required. Other preoperative characteristics, intraoperative data and postoperative outcomes were compared between the groups. Statistical significance was defined as *p*-value < 0.05.

Quality of life was analyzed through the SF-36 subcomponents and component summary dimensions, which were expressed as mean +/− standard error of the mean and 95% CI. The comparison of the groups was performed with the Student’s t-test. To determine further differences between the groups, a principal component analysis (PCA) was conducted for each group, using the scores of the eight subcomponents. The patients from the two groups were represented in a PCA score plot to detect differences in the distribution. All analyses were performed using Stata statistical package v.14 (Stata Corporation LLC). 

## 3. Results

### 3.1. Clinical Characteristics

The comparisons between the two groups regarding demographic and basal characteristics are shown in Table 1. Obesity, defined as BMI ≥ 30, was more frequent in the non-IE group than in the IE group. The pre-operative stroke rates were higher in the IE group compared to the non-IE group (21% vs. 7.6%, *p* = 0.005). The IE patients had lower hematocrit values (31.0 vs. 39.0, *p* < 0.001), higher leucocytes count (8.3 × 109/L vs. 6.86 × 109/L, *p* < 0.001) and higher platelets count (235.0 vs. 198.0, *p* = 0.005). 

Regarding the IE cohort, seventy IE cases were on native valve tissue (66.7%), and the remaining 35 cases had a prosthetic valve (33.3%). There were four patients with negative bacterial cultures, accounting for 3.8% of the IE cohort. Viridans group streptococci (VGS) were the most prevalent causative microorganisms, accounting for 25.7% of the cases. Coagulase-negative staphylococci (CoNS) were the second most prevalent cause of IE (18.1%), followed by Enterococci spp. (13.3%). In our cohort, S. aureus was the fourth leading cause of IE altogether with other streptococci, each representing 10.4% of the cases. *S. gallolyticus* was present in 9.5% of the patients. Our empiric treatment for IE follows the actual recommendations on clinical guidelines [14]. Therefore, most patients undergo treatment with regimens consisting of either cloxacillin + ampicillin + daptomycin or daptomycin + ceftaroline. Once microbiological confirmation either by blood cultures or by cultures of surgical specimens is available, we tailor our antibiotic regimen to a targeted therapy for the causative pathogen for a total of 4 to 6 weeks. 

The surgical procedures are shown in Table 2. No statistical differences were found between the groups regarding cardiopulmonary bypass time and cross-clamp time, although they tended to be longer in the IE group. The valvular substitute was predominantly a tissue prosthetic valve for both mitral (*p* < 0.001) and aortic positions (*p* = 0.007) in the IE patients.

### 3.2. Postoperative Outcomes

Fifty-eight patients (55.2%) of the IE group presented postoperative complications after surgery, compared to forty-six patients (43.8%) of the non-IE group (*p* = 0.129). Postoperative complications included any new atrial arrhythmias, prolonged mechanical ventilation (beyond 48 h), low cardiac output syndrome (cardiac index < 2.2 L/min/m^2^), re-operation for bleeding, AV blockage and need for dialysis. However, only low cardiac output syndrome, need for dialysis and prolonged mechanical ventilation were more common in the IE group in comparison to non-IE group (*p* < 0.05) (Table 3). The patients in the IE group stayed in the ICU a median of 5 days (3–9 days), and the patients in the control group a median of 4 days (3–6 days), but these differences did not reach statistical significance (*p* = 0.051).

### 3.3. Quality of Life

**Figure 1:**
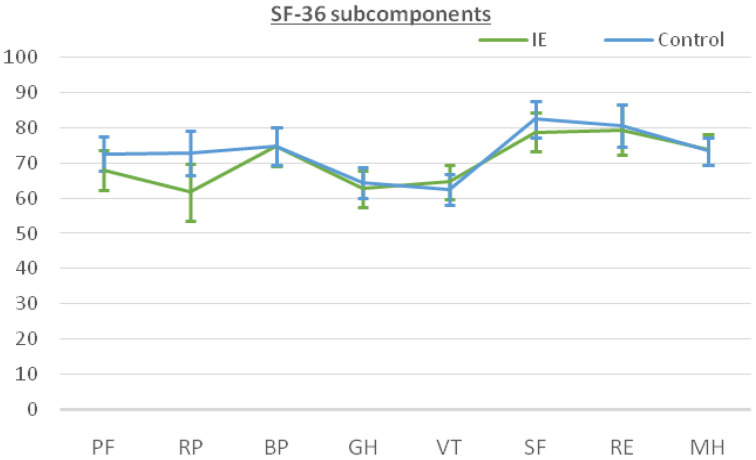
SF-36 subcomponents mean scores and 95% CI for patients subjected to surgery for infective endocarditis (IE, green) compared to patients subjected to cardiac surgery for non-IE purposes (control, blue). Note the overlapping between intervals for each subcomponent, indicating no differences. Abbreviations: PF, physical functioning; RP, role physical; BP, bodily pain; GH, general health; VT, vitality; SF, social functioning; RE, role emotional; MH, mental health.

**Figure 2:**
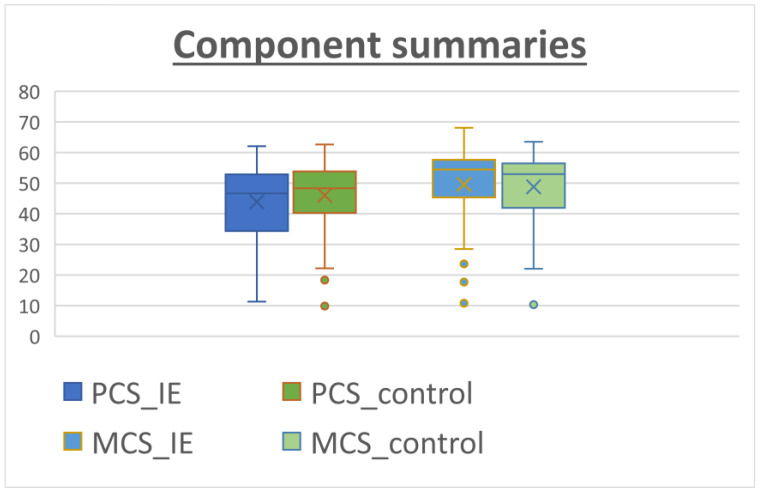
Component summaries for IE patients (blue) and non-IE patients (control, orange). The analysis was performed using relative weights as described by Alonso et al. [18]. The median PCS values tended to be lower than the MCS values.

The patients answered the SF-36 questionnaire after recovery from the cardiac surgery at the last follow-up. The median follow-up time was 33.0 months [22.0–51.0 months] for the IE group versus 43 months [29.0–62.0 months] for the non-IE group. The results for the SF-36 subcomponents are shown in Table 4. The IE patients reported a remarkably high level of perceived QOL, especially in social functioning (SF) and role emotional (RE), with scores of 78.84 and 79.48, respectively. On the other hand, lower scoring values were found for subscales in which the physical component was evaluated such as role physical (RP), general health (GH) and vitality (VT). When comparing the two groups, the patients in the control group tended to perform better in physical subscales rather than in the mental/psychological subcomponents. However, these differences did not reach statistical significance (Figure 1). Figure 2 shows similar results for both groups when calculating physical (PCS) and mental (MCS) subcomponents using the prior PCA analysis described by Vilagut et al. for the general Spanish population [17]. The median PCS scoring in the IE group and control group was 44.26 (42.08–46.44) and 46.33 (44.41–48.24), respectively. The MCS scoring was 49.86 (47.56–52.16) for the IE group and 49.18 (47.16–51.2) for the control group. 

**Figure 3:**
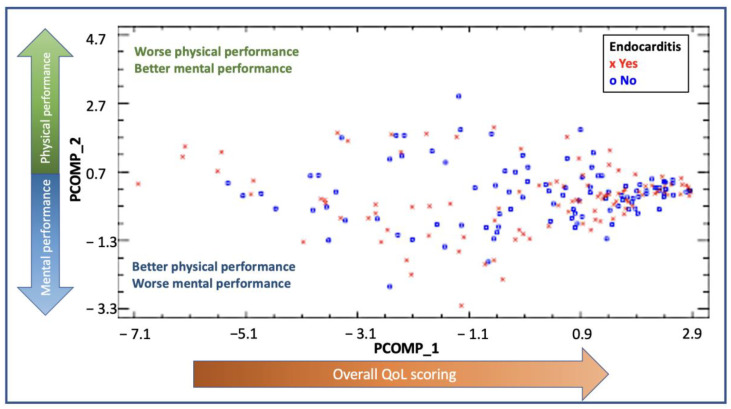
PCA representing the distribution of the cases for the endocarditis group (red dots) and the control group (blue dots). The x-axis represents PCOMP_1, and the y-axis represents PCOMP_2. PCOMP_1 represents the overall quality of life. Hence, patients who perform better on quality of life and have high scores in the SF-36 questionnaire will have higher values of PCOM_1 and will be located on the right end of the Figure. The positive values of P_COMP2 represent a predominant better scoring on physical subcomponents rather than on mental subcomponents. On the other hand, patients who perform better on the mental subcomponents will be located on the lower end of the Figure. Those patients with similar performance in mental and physical subcomponents will be found in the center of the figure. A similar distribution of the dots between the groups is shown, with most dots localized in the right middle part of the distribution, reflecting a better overall quality of life, with a balanced distribution in scoring between mental and physical subcomponents.

The PCA analysis and the relative weights for each of the subscales are shown in Supplementary Table S1. The new components were described as principal component 1 (PCOMP_1) and principal component 2 (PCOMP_2) and explained 73.46% of the variability found in our cohort. In PCOMP_1, all variables had a weight of approximately 0.35, summarizing, hence, the overall quality of life scoring (both physically and mentally). In PCOMP_2, PF, PR, BP and GH had a positive weight, whereas VT, SF, ER and MH had a negative weight or were very close to 0. According to these results, the higher values of PCOMP_2 reflected a better physical performance and a worse mental status. On the contrary, the lower values of PCOMP_2 reflected a better mental status and a worse physical performance. In Figure 3, the distribution by PCOMP_1 and PCOMP_2 of IE cases and controls is shown. The distribution shows high values for PCOMP_1, which represents a high overall QoL, and central values for PCOMP_2, which indicates an overall similar performance of the physical and the mental subscales. Both groups had a similar distribution, reflecting no differences between the groups regarding QoL.

## 4. Discussion

The goals of cardiac surgery in the setting of acute IE are preventing further systemic damage, facilitating cardiac tissue sterilization and reestablishing the hemodynamic function [14]. This should be accompanied by both reasonable follow-up survival and quality of life (QoL). Beyond perioperative survival prediction, a better understanding of the resulting QoL may help physicians, patients and families when facing the decision to consider surgery for IE [7,8,9].

Our cohort included 105 patients, a considerably higher number of patients compared to those in previous studies in which health-related QoL was assessed after surgery for IE [7,8]. The patients with IE experienced more advanced cardiac symptoms and a higher need for emergency treatment. As one might expect, the proportion of preoperative stroke was significantly higher in the IE group (21% vs. 7.6%, *p* = 0.005). The inherent pathophysiology of IE, in which there is an increased risk of systemic embolism, can account for the development of neurological disorders and it has been demonstrated to have a direct impact on morbidity and mortality [19]. Other studies have suggested that the presence of a previous ischemic stroke did not increase the risk of mortality or neurologic complications when surgery was performed early [20,21]. A recent preoperative cerebral embolism could be perceived as negatively influencing QoL after surgery and therefore affect our comparisons. However, in our study, it was not translated into a worse QoL performance at follow-up. Lower hematocrit levels and associated higher leukocytes and platelets levels were found in the IE group. IE represents a pro-inflammatory state with cytokine and acute-phase reactants release, therefore explaining the differences observed between the groups. This inflammatory state has repeatedly been associated with a poorer prognosis, not only in the IE subset [22,23]. Altogether, these factors may explain the higher EuroScore-II values for the IE group, with a predicted higher mortality risk than for the control group. Thus, the acuity of the disease process leading to surgery differs for the two groups. 

Our intention was to assess the quality of life in both groups once a reasonable period of recovery had occurred. Of notice, the median follow-up time for the IE group was 33 months (22.0–51.0 months), compared to 43 months (29.0–62.0 months) for the control group. Slight differences in the moment of obtaining the QoL surveys may be judged as a limitation of our work. However, from a clinical standpoint, we believe that judging the clinical status of these patients on the ground of small differences in the follow-up time should not invalidate the comparison. In fact, the patients with IE had experienced less recovery time at the follow-up assessment. Several reports suggest a sustained long-term increase in QoL after cardiac surgery, which peaks at 1 year after surgery [24,25]. In general, a period of 1 year should suffice to achieve a full recovery from cardiac surgery to judge the outcome of an operation regarding QoL assessment. 

The debate on the choice of the valve substitute for acute IE patients remains wide open. Although recent studies suggested an increased risk of IE after replacement with bioprosthetic valves compared to mechanical valves, we found that patients in the IE group were more prone to receive valve replacement with tissue valves rather than with mechanical valves compared to the control group [26]. Recent studies have demonstrated no differences in survival, reoperation or reinfection in IE patients receiving bioprosthetic versus IE patients receiving mechanical substitutes [27,28]. In our group, these differences in valve selection were justified by an attempt to avoid postoperative anticoagulation. The rationale lies in minimizing the risk of hemorrhagic conversion arising from preoperative cerebral embolic events in IE patients. The existing data suggest no differences related to the valvular substitutes in postoperative QoL [29,30]. However, younger patients tend to score better after mechanical valve substitution, whereas older patients tend to perform better after receiving bioprosthetic valves [31,32]. Whether the implanted valve impacted the QoL could not be determined in our study.

The overall postoperative complications were similar between the groups (*p* = 0.129). Noticeably, low cardiac output syndrome, dialysis or prolonged mechanical ventilation were more common in the IE group, suggesting a different baseline situation and higher acuity in the early postoperative phase. However, these differences were not translated into a worse postoperative QoL after full recovery. Interestingly, the PCS scoring was lower than the MCS scoring, in both groups. These findings suggest that physical performance was more affected than mental status after cardiac surgery. Studies in the general Spanish population described a steeper decline in physical performance compared to mental performance over time [33]. This might align with our observations. Our findings in PCS and MCS performance are comparable to those in the age-matched general Spanish population, i.e., 44.26 vs. 44.82 and 49.86 vs. 49.7, respectively [17]. Regional or national differences may exist. For instance, Perrotta et al. found differences in almost every subcomponent when comparing patients to the healthy population in Sweden [8]. This bigger impact over physical components has also been described in the Dutch population [7]. Further studies are needed to fully understand the long-term impact of IE in comparison to the general population.

We believe the quality of life assessment in survivors of surgery for acute IE is a step forward in understanding the impact of the disease and a marker of the care we provide. There is limited appraisal in the literature for QoL after cardiac surgery and even less after IE. We recognize all the limitations of this non-randomized study. The first and most important limitation of our work is the selection of patients in both groups that survived long enough to answer the follow-up questionnaires. This is, however, a limitation of any study aiming at QoL assessment after an invasive procedure. Another important limitation is the matching itself, as we did not find adequate controls for a subgroup of seven patients with extreme surgical complexity from a technical standpoint. However, this is a contemporary IE surgical cohort experience compared to patients that received operations with reasonable similarity. We aimed to match patients regarding age, sex and surgical procedure; our intention was to include a more commonly seen population of non-IE patients as a reference for clinical comparison and therefore exclude those patients with emergent pathologies in which the core problem is not a valvular disease itself, such as patients with aortic dissection or myocardial infarction. Authors acknowledge the matching process for IE endocarditis patients who undergo surgery is challenging due to the acute nature of the disease. Nevertheless, we aimed to compare IE patients to a group of patients whose indications for cardiac surgery were standard valvular surgery, excluding “outliers” in terms of technical details or infrequent modes of presentation. By doing so, the authors accepted to match the IE patients to a potentially less complex population. Concomitantly, this report may also help in resource allocation and avoid disease discrimination within healthcare systems. 

## 5. Conclusions

Left-sided acute IE patients who undergo cardiac surgery in the acute phase of the disease represent a subset of high-risk surgical patients with an increased rate of postoperative complications compared to non-IE patients who undergo cardiac surgery. Once they recovered from the immediate postoperative period, the QoL status at follow-up appeared comparable to that of patients who underwent equivalent valvular cardiac operations for non-IE purposes. The results of this study support that beyond survival, QoL after surgery for IE justifies the operative management when indicated. 

## Figures and Tables

**Table 1:** Demographic and clinical basal characteristics of patients subjected to cardiac surgery for non-IE purposes (non-IE) and patients subjected to surgery for left-sided infective endocarditis (IE).

Variable	IE (N = 105)	Non-IE (N = 105)	*p*-Value
Age, years	66.0 (55.0–72.0)	66.0 (57.0–72.0)	0.999
Gender			1.000
Male	80 (76.2%)	80 (76.2%)	
Female	25 (23.8%)	25 (23.8%)	
Height, cm	170.0 (164.0–175.5)	179.0 (163.0–175.0)	0.922
Weight, kg	75.0 (68.0–83.5)	80.0 (70.0–88.0)	0.018
Obesity (BMI > 30)	18 (17.1%)	33 (31.4%)	0.015
Smoke	48 (45.7%)	56 (53.3%)	0.269
Diabetes mellitus	25 (23.8%)	17 (16.2%)	0.167
Hypertension	67 (63.8%)	69 (65.7%)	0.773
Hypercholesterolemia	50 (47.6%)	56 (53.3%)	0.408
COPD	13 (12.4%)	17 (16.2%)	0.430
Hepatic disease	8 (7.6%)	2 (1.9%)	0.051
Stroke	22 (21.0%)	8 (7.6%)	0.005
HIV	1 (0.95%)	0 (0%)	0.316
Hepatitis C	3 (2.8%)	2 (1.9%)	0.651
Laboratory parameters			
Creatinine	0.90 (0.74–1.37)	0.95 (0.8–1.1)	0.745
Hematocrit	31.0 (29.0–36.0)	39.0 (37.0–43.0)	0.000
Leukocytes	8.31 (6.88–10.55)	6.86 (5.48–8.31)	0.000
Platelets	235.0 (166.0–310.0)	198.0 (153.0–227.0)	0.005
NYHA IV	40 (38.1%)	8 (7.6%)	<0.001
EuroScore-II	12.23 (4.61–26.51)	3.00 (1.68–6.35)	<0.001
Echocardiographic data			
LVEDD, cm	5.4 (4.9–5.9)	5.5 (5.1–6.25)	0.103
LVESD, cm	3.4 (2.9–4.2)	3.7 (3.1–4.4)	0.028
LAD Ap, cm	4.3 (3.8–4.8)	4.5 (3.9–5.0)	0.061
LVEF, %	60.0 (50.0–60.0)	55.0 (45.0–60.0)	0.213
PAPs, mmHg	40.0 (31.0–55.0)	35.0 (24.0–45.0)	0.002

Abbreviations: BMI, body mass index; COPD, chronic obstructive pulmonary disease; NYHA, New York Heart Association functional classification.; LVEDD left ventricular end-diastolic diameter; LVESD, left ventricular end-systolic diameter; LAD Ap: anteroposterior left atrium diameter; LVEF, left ventricular ejection fraction; PAPs, systolic pulmonary arterial pressure.

**Table 2:** Perioperative characteristics of patients who underwent cardiac surgery for non-IE purposes (non-IE) and patients subjected to surgery for left-sided infective endocarditis (IE).

Variable	IE (N = 105)	Non-IE (N = 105)	*p*-Value
CPB time, min	103.0 (70.0–150.0)	95.0 (77.0–141.0)	0.794
Cross-clamp time, min	78.0 (53.0–120.0)	78.5 (61.0–108.0)	0.889
Aortic valve intervention	77 (73.3%)	78 (74.3%)	0.88
Aortic substitute			<0.001
Bioprosthetic valve	50 (64.9%)	37 (47.4%)	
Mechanical valve	13 (16.9%)	40 (51.3%)	
Homograft	14 (18.2%)	1 (1.3%)	0.922
Mitral valve intervention	53 (50.5%)	50 (47.6%)	0.68
Mitral substitute			0.007
Bioprosthetic valve	28 (70%)	11 (35.5%)	
Mechanical valve	12 (30%)	20 (64.5%)	
Mitral valve repair	13 (12.4%)	19 (18.1%)	
Tricuspid surgery	7 (6.7%)	6 (5.7%)	0.774
Multivalvular surgery	32 (30.5%)	29 (27.6%)	0.648
Aortic root replacement	20 (19.0%)	16 (15.2%)	0.464
Removal pacing leads	4 (3.8%)	0 (0%)	0.127
Previous cardiac surgery	35 (33.3%)	23 (21.9%)	0.101

Abbreviations: CPB time, cardiopulmonary bypass time.

**Table 3:** Post-operative complications in patients subjected to cardiac surgery for non-IE purposes (non-IE) and patients subjected to surgery for infective endocarditis (IE).

Variable	IE (N = 105)	Non-IE (N = 105)	*p*-Value
LCOS	14 (13.3)	5 (4.8)	0.029
Perioperative MI	5 (4.8)	0 (0)	0.068
Dialysis	11 (10.5)	1 (1.0)	0.007
Stroke	7 (6.7)	4 (3.8)	0.344
Bleeding requiring re-operation	12 (11.4)	5 (4.8)	0.073
Catheter bacteremia	6 (5.7)	1 (1.0)	0.121
Sepsis	3 (2.9)	0 (0)	0.241
Atrial arrhythmia	21 (20.0)	25 (23.8)	0.528
AV block	12 (11.4)	9 (8.6)	0.475
Prolonged mechanical ventilation	17 (16.2)	3 (2.9)	0.002
Heart failure	4 (3.8)	1 (1.0)	0.359
Valve dysfunction	2 (1.9)	2 (1.9)	1.000

Abbreviations: LCOS, low cardiac output syndrome; MI, myocardial infarction; AV block, atrioventricular block.

**Table 4:** Comparison between subscales of the 36-item Short Form Health Questionnaire for patients with IE (n = 105) and controls (n = 105).

	Physical Function (PF)	Role Physical (RP)	Body Pain (BP)	Global Health (GH)	Vitality (VT)	Social Function (SF)	Role Emotional (RE)	Mental Health (MH)
IE	67.98 ± 2.76	61.77 ± 4.05	74.64 ± 2.72	62.67 ± 2.54	64.61 ± 2.39	78.84 ± 2.69	79.48 ± 3.54	73.94 ± 2.19
Non-IE	72.66 ± 2.47	72.85 ± 3.13	74.94 ± 2.66	64.34 ± 2.19	62.52 ± 2.17	82.50 ± 2.6	80.63 ± 2.95	73.37 ± 1.88
*p*-value	0.363	0.104	0.979	0.859	0.335	0.135	0.911	0.536

Scores of SF-36 subcomponents for patients subjected to cardiac surgery for non-IE purposes (non-IE) and patients subjected to surgery for left-sided infective endocarditis (IE). Values are expressed as mean ± standard error of the mean.

## Data Availability

The data underlying this article cannot be shared publicly for the privacy of the individuals that participated in the study. The data will be shared on reasonable request to the corresponding author.

## Supplementary Materials

The following supporting information can be downloaded at: https://www.mdpi.com/article/10.3390/microorganisms11041058/s1, Table S1: Relative weights of the principal component analysis.
