# Supplementary material for: Does Quality of Life in Survivors of Surgery for Acute Left-Sided Infective Endocarditis Differ from Non-Endocarditis Patients?
Source: Microorganisms. 2023 Apr 18;11(4):1058. doi: 10.3390/microorganisms11041058 (PMC10142739; doi:10.3390/microorganisms11041058)
Supplement: Supplementary file 1 [file microorganisms-11-01058-s001.zip › microorganisms-2322869-supplementary.pdf]

Annex

Supplementary Table S1. Relative weights of the principal component analysis.

	PCOMP_1	PCOMP_2
**Physical functioning (PF)**	0.366463	0.0233605
**Role physical (RP)**	0.365922	0.324238
**Body pain (BP)**	0.295466	0.573603
**General health (GH)**	0.37818	0.200957
**Vitality (VT)**	0.379679	-0.0945726
**Social functioning (SF)**	0.354612	0.0068303
**Role emotional (RE)**	0.329187	-0.565321
**Mental health (MH)**	0.348845	-0.443105

Supplementary Table S1. Relative contribution of each of the SF-36 subcomponents to the two new components (PCOMP_1 and PCOMP_2). PCOMP_1 describes overall health and QoL as all the subcomponents have similar relative weights. In contraposition, PCOMP_2 express as positive values subcomponents more related to physical performance and negative values those from psychological/mental aspect. Hence, positive values will represent better physical performance over the mental subcomponent, and vice versa.
